# Augmentative and Alternative Communication (AAC) Advances: A Review of Configurations for Individuals with a Speech Disability

**DOI:** 10.3390/s19081911

**Published:** 2019-04-22

**Authors:** Yasmin Elsahar, Sijung Hu, Kaddour Bouazza-Marouf, David Kerr, Annysa Mansor

**Affiliations:** Wolfson School of Mechanical, Electrical, and Manufacturing Engineering, Loughborough University, Loughborough LE11 3TU, UK; Y.Elsahar@lboro.ac.uk (Y.E.); K.Bouazza-marouf@lboro.ac.uk (K.B.-M.); D.Kerr@lboro.ac.uk (D.K.); annysa.mansor@gmail.com (A.M.)

**Keywords:** augmentative and alternative communication, assistive technologies, sensing modalities, signal processing, voice communication, machine learning, mobile health, speech disability

## Abstract

High-tech augmentative and alternative communication (AAC) methods are on a constant rise; however, the interaction between the user and the assistive technology is still challenged for an optimal user experience centered around the desired activity. This review presents a range of signal sensing and acquisition methods utilized in conjunction with the existing high-tech AAC platforms for individuals with a speech disability, including imaging methods, touch-enabled systems, mechanical and electro-mechanical access, breath-activated methods, and brain–computer interfaces (BCI). The listed AAC sensing modalities are compared in terms of ease of access, affordability, complexity, portability, and typical conversational speeds. A revelation of the associated AAC signal processing, encoding, and retrieval highlights the roles of machine learning (ML) and deep learning (DL) in the development of intelligent AAC solutions. The demands and the affordability of most systems hinder the scale of usage of high-tech AAC. Further research is indeed needed for the development of intelligent AAC applications reducing the associated costs and enhancing the portability of the solutions for a real user’s environment. The consolidation of natural language processing with current solutions also needs to be further explored for the amelioration of the conversational speeds. The recommendations for prospective advances in coming high-tech AAC are addressed in terms of developments to support mobile health communicative applications.

## 1. Introduction

Recent studies show that up to 1% of the world population suffers a degree of speech, language or communication need (SLCN) [1,2]. The loss of speech capabilities associated with extreme forms of paralysis and further medical complications has long been regarded as a barrier between the sufferers and the outside world. Augmentative and alternative communication (AAC) incorporates a wide range of processes that augment, complement, or replace speech of individuals with complex communication needs [3,4]. In the broad context of speech and language, *speech* is often associated with the motor movements responsible for the production of spoken words, whereas *language* is associated with the cognitive processing skills of communication.

AAC solutions are classified into three categories: no-tech, low-tech, and high-tech AAC [4]. No-tech AAC is considered the oldest of the three AAC categories, given its reliance on the interpretation of facial expressions and voluntary motor movements, such as sign language, to deliver non-verbal messages [5]. Low-tech AAC utilizes basic tools, such as books and display boards with extended lexicons of images and phrases to aid the communication process [6]. High-tech AAC encompasses the use of electronic devices to achieve an AAC target. Devices falling under this category, such as smart devices and dedicated AAC devices, integrate hardware and software to support a user’s communication needs. A common attribute of modern day AAC solutions tends to rely on the translation of a user’s intended meanings into speech via speech generating devices (SGDs) [4]. AAC communication is also often classified as either un-aided or aided, given the dependence of the solution on the human body solely or the interaction with an external communicative aid for communication, respectively [4].

The potential of AAC intervention has hence been substantial over the last 30 years, with the provision of innovative solutions to a wide range of users with a speech disability [7]. However, although high-tech AAC systems are rapidly evolving, several considerations are yet pertinent to the provision of effective solutions efficiently serving AAC users [4,8]. Low-tech AAC solutions are usually the first techniques tried by speech and language therapists, as the use of simplistic display boards and communication books is both cost-effective and easy to obtain. Moreover, the high costs and complicated training a user requires to operate most high-tech AAC devices could hinder the access to high-tech AAC, and thus the usability of speech generating devices. In turn, an optimized use of high-tech AAC should be researched to provide a faster means of communication, in comparison to low-tech, by prioritizing the communicative needs of the users over the needs of the system. Studies also show that, after testing several AAC systems, the potential of AAC might be limited by complex operational difficulties given the number of users who are simultaneously physically impaired and speech-disabled [8]. Predominantly, AAC users still use combinations of unaided low-tech methods together with an aided high-tech device as suitable for the context of usage and the person they are conversing with [9].

Due to the complex composition of the human body, speech and communication impairments requiring an AAC intervention could result from diverse medical conditions [10,11]. These commonly include Autistic Spectrum Disorders (ASD), strokes, learning disabilities, Locked-in-Syndrome (LIS), Dementia, head and neck cancers, and brain injuries. This also expands to include patients with progressive diseases, such as Parkinson’s disease and Amyotrophic Lateral Sclerosis (ALS) [10]. Other AAC users include patients in transient post-operative states where interventions and treatments, such as ventilator support, may render them unable to speak normally, or at all. In turn, the users benefiting from AAC intervention could be classified into three major groups based on their individual conditions and the intended target use of the AAC communicative aid [12]. These three classes comprise alternative-language users, augmentative-language users, and temporary AAC users. Alternative-language users have a well-established cognitive understanding of language and speech, but have difficulties in conversing. On the other hand, augmentative-language users have difficulties both in understanding speech and in conversing. To be able to use an AAC device, augmentative-language users need assistance in the re-categorization of their surroundings into labels and symbols they comprehend to form a communication language. Temporary AAC users require AAC intervention only for a limited duration of time. This category primarily includes children with developmental conditions, and adults who require transient speech assistance following surgical intervention [12].

Given the complexity of the user base, and the wide need for AAC solutions to serve diverse groups of individuals with a speech disability, current research efforts are being redirected towards the establishment of assistive systems that are suited to respond to their personal users’ needs and capabilities. The aim of this paper is to review the access and processing techniques pertaining to predominant high-tech AAC methods, including the input signal sources, and the developments of machine learning (ML) and deep learning (DL) associated with AAC solutions for the provision of a personalized user experience. This review comprises six sections. Section 2 highlights the relationship between the ACC user needs and the technological developments. In Section 3, the high-tech AAC sensing modalities are classified and reviewed according to their signal sensing sources. A discussion of the listed modalities and a comparison framework of the reviewed systems is presented in Section 4. Section 5 summarizes the discussed findings and concludes the paper. In Section 6, the recommendations for future research are presented.

## 2. Human Interaction

**Figure 1:**
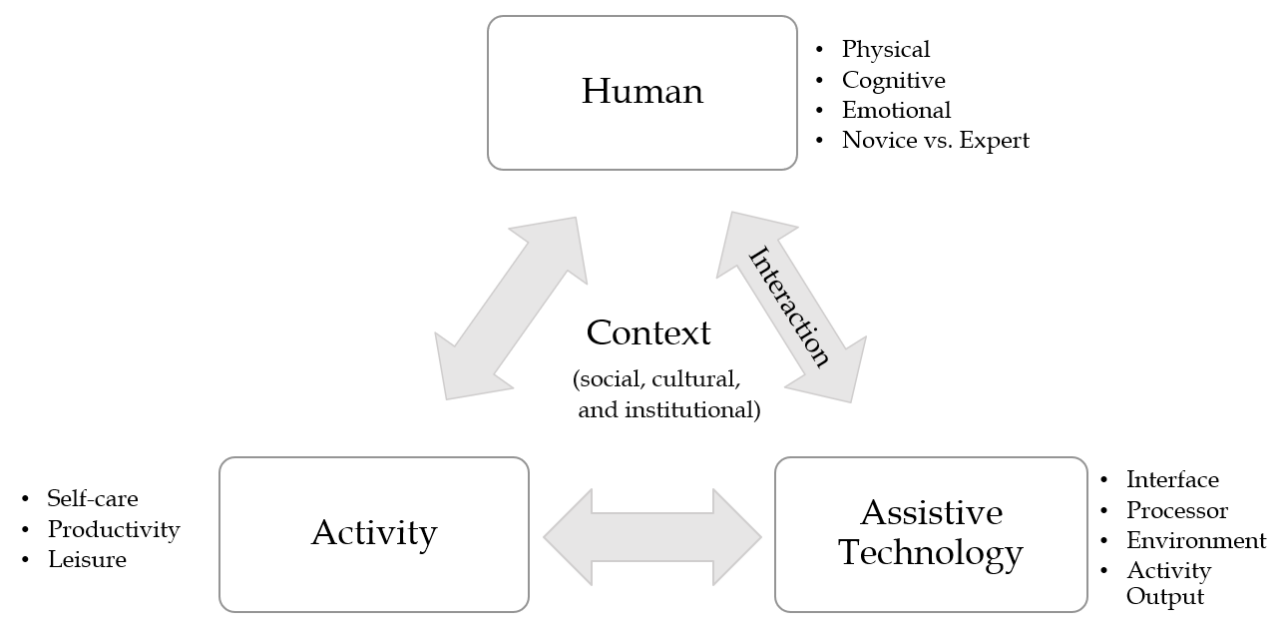
The four components of the Human Activity Assistive Technology (HAAT) model presented in [4]. The interaction between the human and the assistive technology (AT) is emphasized to highlight the relationship between the needs of the AAC users and the elements of development of high-tech solutions discussed in this review.

Several studies exist in the literature of modeling the user’s interaction with assistive technologies (AT) [13]. A primary, well-established, AT framework is the Human Activity Assistive Technology (HAAT) model [4]. The HAAT model underpins a consolidated approach of the interactions between the activity, the human, the context, and the assistive technology. It links the process of selection of an assistive technology solution with the person carrying out an activity in a given context [14]. The four components constituting the HAAT model are shown in Figure 1. Particular attention is drawn to each component, detailing the importance of firstly considering the target activity (self-care, productivity, and leisure), the human abilities of the person using the device (physical, cognitive, emotional, and expertise), the context the device is used in (physical, cultural, and institutional), and in turn the consideration of the suitable AT device (interfaces, processor, and output) [4]. The developers of the HAAT model emphasize on the concept of serving the needs of the users to optimize the usage of the technology, stating that the technology aspect should encompass the function it serves, the person who will be using the AT device, and the context of usage [4].

In light of the HAAT model, AT could hence be used to aid the communication process of individuals with a speech disability, given that the technology prioritizes the activities and abilities of the user. Basing high-tech AAC applications and platforms on the skills and communicative needs of the users, persons with a disability could in turn be allowed to participate in a wider range of activities to communicate their individual needs [15]. From the societal perspective, smart devices have been promoting both the visibility and acceptance of AAC [16]. A number of factors also aids in increasing the access to high-tech AAC platforms, including the ease of operating and using the AAC device, its processing capabilities, the cost of the hardware, and the licensed software packages used to operate the devices [4]. Emphasis is also placed in several studies [4,17] on the importance of customizing AT solutions to address the needs of the users who might find difficulties in accessing the devices’ interfaces. A survey study in [18] further highlights the importance of the provision of technical support and the time taken by a device to communicate a message.

## 3. Sensing Modalities and Their Functionalities

The integration of smart developments into daily life activities has widened the scope of dedicated and non-dedicated AAC applications [7,19]. A survey of high-tech AAC devices with regards to the signal acquisition, ML, and output generation is presented in this section.

### 3.1. AAC Signal Sources and Associated Processing

AAC interfaces are activated through an array of methods for the detection of human signals generated via body movements, respiration, phonation, or brain activities [4]. The acquisition of AAC signals is accomplished through several modalities. Table 1 outlines the AAC signal sensing categories discussed in this review together with their relevant activation methods. The listed AAC access methods could be used in a stand-alone format or in combination with one another. For example, imaging methods may be combined with touch-activated methods or mechanical switches to provide the users with a multi-modal access using the same device. A commercial example is Tobii Dynavox PCEye Plus, which combines several functionalities including eye tracking and switch access to use a computer screen [20].

#### 3.1.1. Imaging Methods

Imaging methods, such as eye gazing, eye tracking and head-pointing devices, have been widely reported in the literature [21,22,23,24,25,26,27,28,29,30,31]. Eye gaze technologies work using the principle of tracking the eye movements of a user for the determination of the eye gaze direction [24,27]. Several eye tracking methods are commonly used, including video-oculography [32], electro-oculography [33], contact lenses [34], and electromagnetic scleral coils [21,25,30,35,36]. Oculography is involved with the measurement and recording of a user’s eye movements [35]. Video-oculography and electro-oculography use video-based tracking systems and skin surface electrodes, respectively, to track the movements of the eye [25]. In the context of AAC, non-invasive eye tracking methods are better suited to address the daily needs of the users who lack motor abilities [27,29]. Practical methods involve the utilization of non-invasive cameras, an illumination source, image processing algorithms, and speech synthesizers to communicate a user’s message [25,27]. Image data are obtained in video-oculography-operated systems using one or more cameras [23,27]. Typical video-oculography systems use glints produced on the surface of the eye through an illumination source, such as near-infrared (NIR) LEDs with typical wavelengths of 850±30 nm, and in turn, gaze locations are estimated from the movement of the eye pupil in relation to the illuminated glint positions [34].

**Figure 2:**
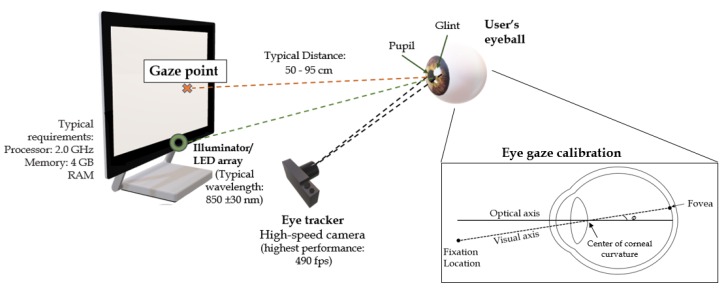
Components of a typical eye gaze system, adapted from [22,38]. The optical and the visual axes are used for the calibration process commonly required to set up the eye gaze system [22,39].

The components of a typical video-based tracking system are shown in Figure 2. Different approaches are presented in the literature for calculating the accuracy of an eye tracking system, including the distance accuracy (in cm or in pixels) and the angular accuracy (in degrees) [22]. The pixel accuracy can be given by

(1) $$P_{acc}=\sqrt{{\left(X_{target}PX\right)}^{2}+{\left(Y_{target}PY\right)}^{2}}$$

where $X_{target}$ and $Y_{target}$ are the coordinates of the target points, and PX and PY are the gaze point coordinates given by

(2) $$PX=mean\left(\frac{PX_{left}+PX_{right}}{2}\right)$$

and

(3) $$PY=mean\left(\frac{PY_{left}+PY_{right}}{2}\right)$$

respectively, with the subscripts left and right referring to the coordinates of gaze points of the left and right eyes. The on-screen distance accuracy (DA) is similarly given by

(4) $$DA=p_{size}\sqrt{{\left(PX-\frac{x_{pixels}}{2}\right)}^{2}+{\left(y_{pixels}-PY+\frac{offset}{pixelsize}\right)}^{2}}$$

where $p_{size}$ is calculated based on the resolution, height, and width of the screen, $x_{pixels}$ and $y_{pixels}$ are the pixel shifts in the directions of x and y, respectively, and the offset is the distance between the eye tracking unit and the lower edge of the screen [22,37]. The angular accuracy (AA) can be also computed via

(5) $$AA=\frac{p_{size}\times P_{acc}\times {cos(mean(\theta ))}^{2}}{meandist}$$

where the gaze angle θ is given by

(6) $$\theta =tan^{-1}\left(\frac{DA}{dist}\right)$$

and *dist* and *meandist* are the distances from the eye to the screen and from the eye to the tracker, respectively [22,37].

Fixations and saccades are commonly used to analyze eye movements [40]. Fixations are the pauses a user intently inputs by fixing his eye movements at the target gaze point, whereas saccades are the eye movements rapidly occurring following and in between the fixations. Metrics of eye gaze estimations include fixation durations, fixation rates, fixation sequences, saccadic amplitudes and velocities [22,40]. Although electro-oculography is a cost-effective eye tracking method, Infrared pupil corneal reflection (IR-PCR) video-based systems are most commonly used by speech and language practitioners due to their non-invasive nature [25,27]. A calibration operation is essential in video-based trackers to fine-tune the system with a user’s eye movements [41]. As shown in Figure 2, a user’s visual axis deviates from the optical axis upon the usage of a gaze system. Calibration is expressed as the process of finding the visual axis pertinent to each user by calculating the angle between the line joining the fovea (the highest point of sensitivity in the eye retina) with the center of corneal curvature, and the optical axis [22].

The estimation of the visual axis is usually not feasible, and as such, the calibration process enables the tracker to capture and learn the difference between the user’s eye positions when gazing at a specific target in comparison to the actual coordinates of the gaze target. The user’s head orientation should be also considered in IR-PCR systems, as the movements of the user’s head can adversely impact the calculations of the glint vectors [22]. Studies are however addressing advances in eye tracking methods to overcome the related constraints, providing the forthcoming possibilities of free IR eye tracking and robust algorithms for head movements compensation [42].

A large number of eye tracking and eye gaze AAC applications is commercially available. Several AAC eye gaze and eye tracking applications, such as Tobii Dynavox PCEye Plus [20] and Eyespeak [43], can be accessed in a multimodal form. This enables the users to use other methods of input, such as switch access, headtracking or touchscreens together with the tracking software to suit their individual needs. IntelliGaze (version 5) with integrated communication & environment control [44] is also an example of an eye tracking AAC tool which allows sending and receiving messages for an improved communication. Most of the listed solutions include extensive vocabulary sets, word predictions, and advanced environmental controls for an enhanced support of the user. Other eye tracking systems, such as EagleEyes [45], allow the control of an on-screen cursor via the electrodes placed on the user’s head to aid the communication of users with profound disabilities [31].

#### 3.1.2. Mechanical and Electro-Mechanical Methods

Mechanical and electro-mechanical AAC devices have applications for both direct and indirect selection access methods. Direct selections offer the users sets of choices, and require a voluntary input selection of the intended messages from the user’s side. This usually involves the coordination of voluntary controls using a body part, such as the hand or fingers, or a pointing device, to select a message [19]. Mechanically activated direct-selection methods include mechanical keyboards, which utilize the physical mechanical depression of the pressed keys to activate a user selection. Keyboard layouts maybe reconfigured for individuals who find the use of a standard keyboard difficult due to the required coordination between the two hands [4].

**Figure 3:**
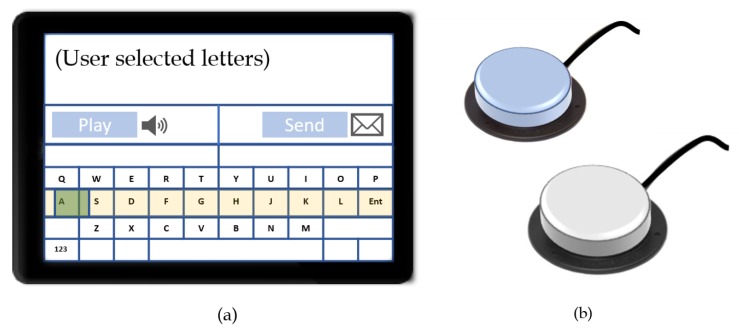
(**a**) A sample visual scanning interface activated via switch scanning. The yellow box moves vertically across the lines until a selection is made, followed by a gliding green box moving horizontally across the highlighted line until a letter is also selected. In (**b**), two scanning button switches are displayed.

For individuals lacking voluntary controls, communication via direct selections is often cumbersome, and, consequently, indirect selection methods are best-suited for this group of users [19]. Scanning methods are predominantly in use with indirect selections, involving a systematic representation of options appearing in timed intervals for the users to select from [19,46]. Mechanical scanning methods include single switches, arrays of switches, or other variations of methods activated via the application of a force [4]. Switches are generally considered a form of low-tech AAC due to their minimal hardware requirements; however, switching applications have recently expanded to allow users the access of several high-tech AAC platforms, including computers, tablets, or smart devices via scanning. Scanning techniques range across three levels, each suited to accommodate users with specific motor abilities: Automatic scanning is used to present items in adjustable time intervals, based on the user’s skills, until a selection is made; step scanning allows the users to control the presentation of selections, in turn controlling the rate of advancement; and inverse scanning involves holding down a control interface and releasing it upon the desired selection [4]. Figure 3 shows a visual scanning interface together with typical activation switches.

In addition to letters, scanning interfaces expand to include a variety of access options, including icons, pre-stored messages, and auditory messages. Some operating systems also provide the option of device navigation via an external switch. The position and access methods of switches are user dependent. They can be adjusted to be in close proximity to the hands or the feet for the ease of activation. Mechanical switches can be also mounted on wheelchairs to allow access using head movements. Different variations of switches are available in terms of shapes and types to suit the user’s requirements. In general, mechanical switch scanning requires minimal motor movements; however, the communicative rates could be slowed down by the delay required to make a selection. Nonetheless, based on the requirements of some AAC user groups, indirect access methods utilizing switch scanning may still aid in the communication of basic needs. As implied from the HAAT model, the user’s requirements specify the objectives of using a communication aid. Therefore, the independent communication of these user groups could be among the primary targets of using an intervention.

#### 3.1.3. Touch-Activated Systems

With the escalation of the touchscreen developments, touch-activated AAC applications are commonly in use with AAC direct selection activation. Touchscreen technologies comprise various types, including resistive, capacitive, surface acoustic wave, and optical/infrared touchscreens [47]. Resistive and capacitive touchscreens are predominantly used with smart devices [48]. Resistive touchscreens are dependent on the production of a force or pressure using the user’s fingers, whereas capacitive touchscreens are activated using the electrical charge present on the user’s finger [49]. Although resistive touchscreens are cost efficient, capacitive touchscreens are often known to present a better visual clarity, presenting an added benefit for AAC users suffering a degree of visual impairments. Touch membrane keyboards are also in use by AAC users. They are built using non-conductive spacers separating conductive flat surfaces, and acquire electronic signals through the pressure resulting from holding down a key, generating an input signal to the AAC device [19].

**Figure 4:**
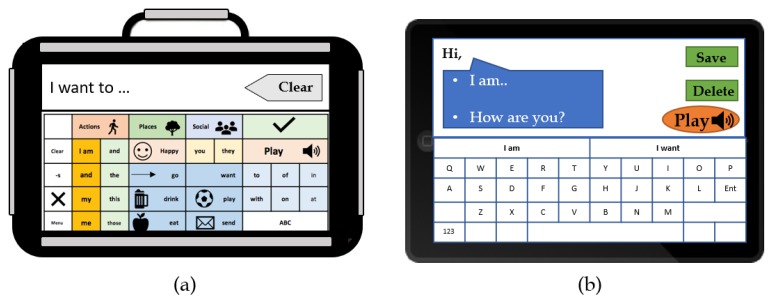
Examples of (**a**) a dedicated touch-based device and (**b**) a non-dedicated smart device running an AAC application (APP), usually with predictive language model and speech generation capabilities.

Several AAC touchscreen applications, such as Verbally [50], Proloquo2Go [51], and PredictableTM [10,52], are currently available for the use with tablets and smart devices for a rapid and portable access to an AAC solution. The tools operate based on a variety of activation methods, primarily including image-based solutions and word spelling for synthesis via the device’s inbuilt text to-speech capabilities and speech generation, as shown in Figure 4. The interfaces of the applications could be usually tailored to allow users the flexibility of setting up the devices according to their needs. The costs of the solutions vary according to several factors, including the capabilities of the tool and the sophistication of the software. AAC users utilize touchscreens and touch activated systems to make selections via swiping and tapping; however, such actions could be restrictive for users who are physically impaired [4]. Nonetheless, the accuracy can be augmented using pointers, as the icons presented on a touchscreen often have the advantage of being cognitively easy to select, and less demanding in comparison to the operation of a regular computer [4].

#### 3.1.4. Breath-Activated Systems

**Figure 5:**
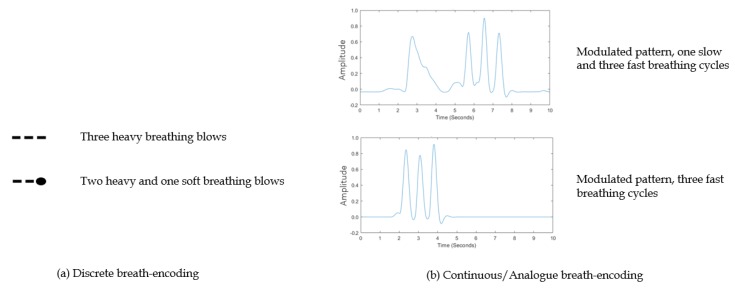
Examples of (**a**) discrete breath encoding, where soft and heavy breathing blows are recorded to encode combinations of zeros and ones, or Morse codes, representing the intended messages, and (**b**) continuous breath encoding, where the speed, amplitude, and phase of breathing are modulated to create patterns representing the intended message.

**Figure 6:**
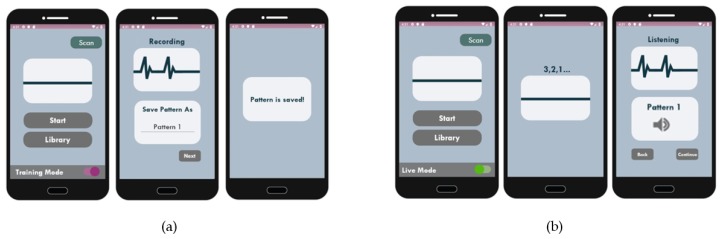
Examples of (**a**) training mode, and (**b**) live mode of continuous breath encoding for the storage and the retrieval of breathing patterns linked to a user phrase using a mobile APP.

The wide availability of sensing modalities expands the scope of AAC control interfaces to include the detection of respiratory signals in addition to the regular voluntary body movements [4]. Voluntary body movements are commonly detected through the integration of sensors with imaging, and/or optical, mechanical, and electro-mechanical devices. Respiration signals are recorded via a wide range of modalities, including fibre optic sensors [53], pressure and thermal sensors [54], photoplethysmogram (PPG) measurements [55], electroencephalogram (EEG) signals [56], and the examination of airflow [56,57]. Discrete and continuous breathing signals can be used to encode messages, as shown in Figure 5. Discrete breath encoding involves the generation of soft and heavy breathing blows encoded as binary combinations of zeros and ones, or Morse codes to represent the user’s intended messages or the International Morse code’s letters, respectively. On the other hand, continuous breath encoding uses the modulation of the speed, amplitude, and phase of breathing signals to create patterns representing the intended message. The modulation of the continuous breathing patterns encoded to represent user selected phrases, including the training and retrieval modes, is shown in Figure 6 for a mobile based APP.

An early respiration activated AAC development involving a breath-to-text application was initiated at the Cavendish Laboratory at Cambridge University [58]. The study presented the use of fine breath tuning to use Dasher to support the communicative requirements of AAC users. Dasher is a text-entry system with a predictive language model available on several operating systems, and uses one- and two-dimensional inputs from pointing devices to access an on-screen cursor. The fine breath tuning system encodes letters using Dasher’s interface and a specially designed thoracic belt worn around the chest. Two inches of the belt are replaced by an elastic material, with a sensor measuring the changes of a user’s waist circumference resulting from breathing variations. The study reports an expert user conversational rate of 15 words per minute using this system. The usage of sniffing signals was also established in the scope of AAC in [59]. A device was developed for the measurement of human nasal pressure via a nasal cannula and a pressure transducer. The device was tested with individuals in LIS, and quadriplegic users. To write text, the captured nasal pressure changes are converted into electrical signals, and passed to a computer. The device comprises two associated interfaces for the user’s selection of letters, including a letter-board interface, and a cursor-based interface. The system aids the users in LIS, with reported rates of three letters per minute.

Microphones could be also used in combination of an AAC interface. The loss of speech abilities associated with SLCN centralizes the usage of microphones around two AAC areas, including speech augmentation of individuals suffering partial loss of speech [60] and breath encoding for individuals with a speech disability [57,61]. Speech augmentation applications, such as “Voiceitt” [62], are currently researched to aid the communication of individuals suffering from Dysarthria or using non-standard forms of speech. “Voiceitt” uses a specialized software and the inbuilt capabilities of a portable device to understand dysarthric speech and allow a real time user communication. On the other hand, breath encoding is being researched to aid the communication of the users lacking speech abilities. Encoding distinct inhalation and exhalation signals is presented in [61] to produce synthesized machine spoken words (SMSW) through soft and heavy blows represented through four-bit combinations of zeros and ones. The classification is achieved based on the threshold values of the generated blows. A micro-controller unit together with an MP3 voice module are appended to the microphone for the execution of the pattern classification and the playback of SMSW. The 16 discrete combinations are linked to predefined phrases selected with the aid of medical practitioners. A device named “TALK” is also a solution involving a micro-electro-mechanical-system (MEMS) microphone together with two low-cost micro-controllers, and is similarly in use with distinct inhalation and exhalation signals to encode letters through the International Morse Code to produce SMSW [2]. A study also reports the use of analog breath encoding for AAC purposes by utilizing the recognition of continuous breathing modulations [57]. Analog encoding of the acquired breathing signals is reported to provide an increased bandwidth at the low breathing frequencies, as it utilizes the signal’s amplitude, frequency and phase changes to encode a user’s intended meanings. The classification is achieved based on the dynamic time warped distances between the tested breathing patterns. A systematic reliability of 89% is reported with increased familiarity with the system.

#### 3.1.5. Brain–Computer Interface Methods

**Figure 7:**
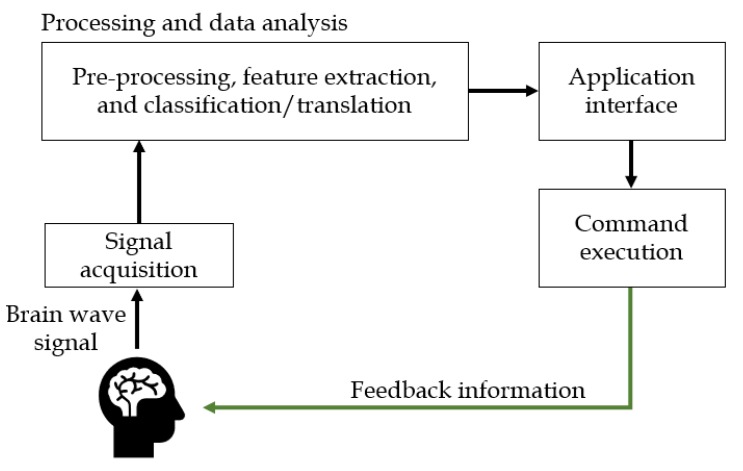
The components and flow diagram of a Brain–Computer Interface (BCI) system, adapted from [66,67].

In the scope of AAC, Brain–Computer Interface (BCI) solutions are being widely researched to allow AAC users to control external devices by modulating their brain signals [63,64,65]. Brain interfaces are either invasive or non-invasive. Invasive interfaces involve the usage of implanted electrodes and the interconnections of the brain with the peripheral nerves [64]. Non-invasive BCIs comprise the usage of external devices to monitor a user’s brain activities through EEG [60,64], magnetoencephalography (MEG) [63], functional magnetic resonance imaging (fMRI) [63,64] or near-infrared spectroscopy (NIRS) [63,64]. The components and flow diagram of a typical BCI system are shown in Figure 7.

EEG is a popular BCI recording method, given its non-invasive nature and its relatively lower cost [68,69]. In electrical BCI systems, the brain produces a set of electrical signals when triggered by a stimulus, known as the evoked potential [70]. EEG signals are acquired using 2–64 sensors placed on the scalp of the user to record the brain activity [71]. Amplifiers and filters are typically utilized, with an output fed back to the user to accordingly modulate the brain activity [64]. To translate a brain activity into a computer command, regression and classification algorithms can be used [72]. An adaptive auto-regressive (AR) parameter estimation model used with EEG BCI describes a time series signal *x(t)* as

(7) $$x\left(t\right)=\sum_{i=1}^{p}\phi_{i}x\left(t-i\right)+\epsilon_{t}$$

(8) $$x\left(t\right)=\phi_{1}x\left(t-1\right)+\ldots +\phi_{p}x\left(t-p\right)+\epsilon_{t},$$

where $\phi_{i}$ and p are the AR coefficients and the order of the model, respectively, and $\epsilon_{t}$ is white noise [73,74]. A review study [72] demonstrates that the use of classification algorithms is an increasingly popular approach with BCI interfaces, as they are commonly used to identify the acquired brain patterns. Classification is the process of using a mapping *f* to predict the correct label *y* corresponding to a feature vector *x*. A training set *T* is used with the classification model to find the best mapping denoted by f* [72]. The classification accuracy of a model is dependent on a variety of factors. A study [72] demonstrates that, using the mean square error (MSE), three sources are identified to be the cause of classification errors, given that

(9) $$MSE=E[{\left(y-f\left(x\right)\right)}^{2}]$$

could be decomposed into

(10) $$MSE=Var\left(f\left(x\right)\right)+Bias{\left(f\left(x\right)\right)}^{2}+\sigma^{2},$$

where the variance *(Var)* represents the model’s sensitivity to *T*, the *Bias* represents the accuracy of the mapping *f*, and the noise $\sigma^{2}$ is the irreducible error present in the system. Common ML algorithms used with BCI include linear classifiers (such as linear support vector machines), neural networks, nonlinear Bayesian classifiers, nearest neighbors, and combinations of classifiers [71,72]. Signal processing techniques pertinent to BCI methods include both time-frequency analysis, such as AR models, wavelets, and Kalman filtering, and spatiotemporal analysis, such as the Laplacian filter [75]. Hybrid BCI is a different approach to brain signals processing, combining a variety of brain and body signals in sequential and parallel processing operations with the aim of improving the accuracy of BCI systems [76].

BCIs are under continuous research to aid the communication of individuals suffering from motor strokes [63], ALS, and LIS, and spinal cord injuries [77]. BCI systems involve three basic pillars, including the user training, the associated ML, and the application in use [78,79]. Research in the area of BCIs is currently evolving [63], with promising results in recent state-of-the-art projects. A study by Stanford University [80] confirmed the usability of BCIs to control an unmodified smart device for quadriplegic users. BCIs have been also in use to surf the Internet [81], with an EEG BCI based application tested with LIS and ALS conditions [82]. It is also reported that BCIs could aid users control spelling and play games [80].

### 3.2. Machine and Deep Learning

Typical signal processing of the acquired AAC signals encompasses three primary operations: encoding, prediction, and retrieval [10]. Encoding involves the conversion of the acquired signal into a pre-defined format accepted by the system for the production of a specified output, whereas prediction is concerned with building the algorithms used to select the desired output [10]. Prediction encompasses several operational contexts, including word [83], message, and icon prediction [10]. In general, an ideal AAC system should integrate self-learning capabilities to respond to its users’ individual needs [2,8]. Demographic data show that current AAC users belong to numerous cultural and linguistic backgrounds [7]. In turn, the design of systems tailored to address specific users’ requirements is vital for an enhanced adaptability. High-tech AAC is hence becoming a highly interdisciplinary area of research, combining rehabilitation engineering with clinical and psychological studies, signal processing, and ML [84].

ML has been widely evolving over the last decade, with a number of applications aimed at aiding the provision of intelligent AAC solutions to address the users’ needs. The automation of algorithms, prediction, and classification capabilities presented by ML solutions could be of great benefit to the users. Technologies such as natural language processing (NLP) are highly dependent on artificial intelligence (AI). The operation of NLP is centered around the analysis, augmentation, and generation of language, including the computation of probabilities of incoming words and phrases, and complete sentence transformations [85]. NLP has various applications in AAC, utilizing ML and statistical language models to process and generate outputs by optimizing word prediction models, topic models [86], speech recognition algorithms, and processing of the context of usage [85]. BCI is also highly dependent on ML, as users learn to encode the desired intended messages through dedicated brain signal features captured by the BCI for the translation to the intended meaning or the desired control [78,84,87,88]. Recent studies also show that advances with DL algorithms, such as conventional and recurrent neural networks, could have a potential superior performance in comparison with conventional classification methods [70]. As demonstrated in [89], ML is also used in conjunction with ECG spelling based BCI applications to minimize training times, although the conversational rates are still generally reported to be slow [90]. On the broader scale, research in [91] demonstrates that Neural Networks could be potentially used to learn, predict, and adapt to the events within a user’s environment to aid the people with disabilities.

### 3.3. Outputs and Speech Generating Devices

High-tech AAC systems can produce outputs in a variety of formats, including symbols, icons, and electronic digitized or synthesized speech [10]. SGDs, or voice output communication aids (VOCAs), are devices with the ability to produce digitized or synthesized speech [9,92]. Digitized speech is pre-stored speech acquired via a microphone and stored in electronic format for retrieval upon a user action [93], whereas synthesized speech is generated based on mathematical algorithms and played as natural voice [10]. The wide availability of smart devices facilitates the access to VOCA applications. Synthesized speech includes the production of output messages via text to speech synthesis, and is therefore commonly researched to assist the communication and free personal expression of individuals with a speech disability. This is primarily due to the benefit of providing a greater flexibility in contrast with digitized speech. Studies show that AAC devices with SGD capabilities contribute to significant developments in terms of AAC solutions [10,92]. However, the efficiency and effectiveness of using a VOCA with an AAC user remains dependent on the user’s abilities, their medical condition, and the communication partners they are conversing with [9].

## 4. Comparison of Existing AAC Signal Sensing Methods

With respect to the discussed HAAT model and the listed AAC access methods, the integration of state-of-the-art AAC systems with AI applications could help in the improvement and the ease of use of common AAC devices and their associated user interfaces. The focus on the user activity to be carried out needs to be at the core of the implementation. Table 2 provides a comparison of the input signal sources, the requirements for operation, the areas of strength, and the areas of limitation of the listed AAC sensing methods. A summary of each of the listed access categories is demonstrated below in terms of the ease of access, affordability, ease of programming and maintaining, portability, and conversational rates.

### 4.1. Ease of Access

Imaging methods, including eye gaze and eye tracking methods, are generally utilized as non-invasive means of communication for the individuals with minimal voluntary controls and motor movements. The natural eye gazing process is a an advantageous trait for accessing devices [26]. However, typical imaging methods were shown to require a learning curve for both the users and the systems, as a calibration step is usually required for the customization of an imaging device to each individual user [94]. The accuracy of the system is also dependent on many variants, including the gaze angle, the pixel accuracy, and the distance between the eye and the screen, as demonstrated in Equations (1), (4), and (5), rendering eye gaze difficult when selecting small items on the screen [26]. The movements of the head and the direction of the gaze might impact the usability of the acquired signals, usually with algorithms implemented to cancel out the effects of such movements [22]. Recent eye gaze systems are better proofed against head movements, and in turn need to be calibrated less frequently [60]. Calibration models are also in use to facilitate the process of gaze calibration [95]. Recent studies are beginning to address the current constraints to create forthcoming robust imaging systems that are easy to use [94]. Mechanical and electro-mechanical activated switches and keyboards are usually easier to operate due to their simplistic nature. Mechanical switches are predominantly used with individuals requiring minimal motor movements to access a computer or a smart device via indirect selection [4]. Touch-activated methods require voluntary muscle controls, however with a minimal activation pressure, as discussed in Table 2. Touchscreens can also be used in combination with mechanical switches for individuals lacking motor controls to access the devices for indirect selections enablement. This multi-modal access can in turn be advantageous, as users will have a choice to access the device using more than one modality. Breath-activated methods are similarly used with individuals with minimal voluntary controls; however, they require a training step to recognize the selected patterns [57,61]. With regards to BCI access, non-invasive methods, such as EEG, are used due to their non-intrusive nature. BCI systems provide a natural means of access, aiding the users to gain independence [96]. However, the signal acquisition from the brain is at times cumbersome for the users, especially with EEG applications requiring the use of electrolytic gel to facilitate the acquisition of the brain signals from the scalp [97]. The length of the training process required to use a BCI system could also present a challenge for usage [98].

### 4.2. Affordability

In terms of costs, the expenses associated with the hardware and software requirements of the utilized platforms directly impact the expenses related to the systems. Imaging methods, including eye gazing and tracking, are relatively expensive in comparison to switch access, touch-based methods, and breath-activated methods. This is mainly due to the high-costs associated with the systems’ hardware requirements, which are listed in Table 2, together with the costs of research, programming and maintaining the devices [99]. Depending on a solution’s capabilities, the price of a typical eye tracker ranges from hundreds to thousands of dollars [94]. Some solutions are emerging to reduce the costs of imaging AAC devices [27,94]; however, more research is still needed to widen the scope of usage of highly performing, low-cost eye trackers. On the other hand, mechanical keyboards and access switches are commonly simple to design and thus they are usually more affordable. The reliance of switch access or touch-based methods on a smart or a high-tech dedicated device could increase the costs of the provided solutions; however, with the prevalence of smart devices, several AAC communicative applications (APPs) are now available on various operating systems, widening the usability of AAC in contrast with traditional SGDs [16,100]. As listed in Table 2, breath-activated methods are usually accessed using pressure sensors or microphones together with micro-controller boards or a computer. The hardware requirements could increase the costs of the solutions; however, the escalating prevalence of smart devices might aid in the provision of cost-effective breath-activated APPs. BCI methods are also being researched to reduce the costs associated with the systems [76]; however, low-cost BCI systems are reported to require further research to improve the accuracy and quality of the acquisition in comparison with advanced BCI systems.

### 4.3. Ease of Programming and Maintaining

Programming an AAC access modality is dependent on the acquired signals, together with the research and skills required to set up and maintain the systems. Typical imaging devices are associated with increased complexities in terms of algorithm writing, data processing, and data parsing [101,102]. This in turn requires extensive programming and coding skills to set up the gaze detection algorithms, calibrate the sensors to individual users, and accurately respond to the needs of the users. The resulting data also need to be addressed, with specific considerations to sample sizes and data resolution [102]. Mechanical switch access of smart devices, and the programming of touch-activated APPs is in turn less variant in terms of calibration and set up. The APPs however need to be carefully designed and tested to respond to the user’s input generated via a switch, a keyboard, or a touchscreen. Touch-activated methods also need to incorporate a visual or auditory feedback mechanism to confirm the user’s selection, as demonstrated in Table 2 [19]. Breath-activated methods are similarly programmed based on breath thresholds [61], and classification algorithms [57]. The complexities are in turn dependent on the requirements of the APP design together with the selected classification algorithms required for the system operation. Concerning BCI methods, the challenges related to managing and programming the systems are centred around the information transfer rates, the non-linearity of the systems, and the complexities associated with the high signals’ dimensionalities [98].

### 4.4. Portability

In terms of portability, the typical requirements of the systems dictate the ease of moving the device for usage in a different setting. Commercial solutions of the AAC imaging methods are starting to address this constraint to increase the usability of the devices [42,94]; however, most typical system requirements still restrict eye-tracking systems to be used indoors [23] or together with a monitor. On a similar note, the portability of mechanically activated switches is variant depending on the context of usage; however, the integration of switch access with mobile and smart devices increases the ease of portability. Touch-activated methods are similarly highly portable, given the typical sizes of the smart devices used in coordination with the method. On the other hand, the portability of breath-activated systems is application dependent, as the solutions requiring the need of a computer interface still need to be developed to address this constraint. BCI methods are still challenged in terms of the communicative interfaces [98]; however, some advances in BCI have been reported for the potential possibility of home usage [96] and increased portability [103].

### 4.5. Conversational Rates

Natural speech has a rate of 125–185 words per minute (WPM) [104]. Speech rates of less than 100 WPM are identified as slow [105]. Direct selection techniques, including eye gaze systems, are found to provide conversational rates of about 8–10 WPM [104]. Similarly, mechanically activated AAC switches and keyboards also affect the conversational rates. The automatic, step, and inverse activation of switches often requires the users to wait until the desired selection is displayed, introducing conversational delays. Scanning methods are reported to allow communicative rates of around two WPM [104]. Selecting letters to form words may also impact the user’s communication rate. This is apparent in touch-activated methods, where users are required to spell words or select icons to form sentences or to write text. The conversational rates of breath-activated systems are further dependent on the encoding method, as systems where breathing variations are used to select letters to write words could negatively impact the conversational rates. A recent study [97] similarly reports that most BCI technologies still offer conversational rates of less than 20 letters per minute. Generally, the rates of conversation using AAC systems, including word prediction and letter abbreviation, were found to be 12–18 WPM, highly contrasting with the rates of natural speech [104]. For some AAC user groups, such as individuals primarily requiring an independent form expression, the communication of basic needs is at times prioritized over the speed of conversation. Nonetheless, moving beyond transactional conversations remains a target for current state-of-the-art AAC technologies for an enhanced experience when using an AAC tool [104].

## 5. Conclusions

In this review, a global view of predominant high-tech AAC systems is presented in relation to their signal sensing categories, including the modalities’ key features and sensing mechanisms. The listed categories are analyzed in terms of their strengths and limitations to highlight the advantages and drawbacks of the discussed high-tech AAC systems. The review focuses on consolidating the current prevalent AAC tools from the technological aspect to provide a global view of the contemporary interventions.

Considering the comparison framework and the AT requirements of the HAAT model, AAC technologies evidently provide solutions that increase the participation and engagement of individuals with a speech disability. However, such technologies are still generally challenged in terms of an optimal usage centred around the user’s intended activity. The development of robust AAC solutions should consider some of the shortfalls of the current technologies. These primarily include addressing the affordability requirements of most high-tech AAC sensing modalities, as they negatively impact the scale at which high-tech AAC is expanding. This also expands to include the adaptability of the interventions to respond to the different needs and requirements of the user in compliance with the HAAT model. The increased complexities of some AAC systems might also require special support from the user’s carers to set up and operate the systems, and in turn some tools have remained restrictive. AAC solutions are also still confined to approximately 10% the rate of natural speech [110] for narrative speech and conversation.

The on-going high-tech AAC research activities have been consolidated in terms of their sensing modalities to include the conventional AAC works and moving beyond the present existing systems to address the requirements of individuals with a speech disability. Based upon this point, the review emphasizes that the signal source plays a vital role to well understand what flexible AAC devices acquire. The potential of high-tech AAC systems could be foreseen to help in the expansion of the current tools beyond their present applications to include an improved user engagement and advanced unrestricted means of communication.

## 6. Future Research and Prospective Advances in AAC

State-of-the-art AAC tools demonstrate a potential for the provision of an enhanced user experience centred around the needs of the users. Based on the comparison framework and the AAC sensing modes presented in this review, the following implications can be drawn regarding the prospective AAC advances and recommendations for future research.

### 6.1. Signal Sources and Usable Information Content

The facilitation of the signal acquisition and robustness of the control interfaces play a significant role in the general usability of the devices. The comparison of the reviewed modalities for sensing body signals generated via voluntary movements, respiration, phonation, or brain activities demonstrate that the ease of using a solution for effective communication is commonly related to the ease of acquiring the desired signals. Moreover, the usability of the solutions tends to be related to the number of user environments in which the device is fully functional increasing the likelihood of a regular usage of the device. For example, Infrared (IR) based imaging systems and the sensitivity of ECG BCI systems could restrict the usage in specific environments. Continuing to address these constraints could therefore be beneficial in terms of the increased rate of usability of the systems. An unobtrusive use of a communication aid is also critical, and in turn, invasive AAC sensing methods, such as invasive BCI, are usually less frequently used. Similarly, efforts are being directed at reducing the rigorous signal calibration required by imaging systems to facilitate the usage. Future AAC solutions maximizing the use of the sensed signals would hypothetically provide added advantages in terms of ease of control, and in turn the possibility for an improved communication output. Capitalizing the information content of the sensed signal while reducing the activation requirements from the user’s side would therefore theoretically provide an increased bandwidth of usability benefits.

### 6.2. Intelligent AAC, DL functionalities, and NLP

The advances of the integration of state-of-the-art AAC systems with AI and DL applications could be researched to further improve the access to high-tech devices, the speed of the output generation and the customization and adaptability of the AAC interfaces to suit the needs and requirements of each individual user. Recent research also reveals a direction of adopting DL in assistive communication applications by recognizing and anticipating the user’s environment [111]. For narrative communication applications, pre-programmed phrases restrict the dynamicity of the user’s conversations, whereas spelling-based communication is generally slow. Further research with regards to NLP and DL functionalities is needed for the provision of innovative activity-oriented AAC methods to support the user, the facilitator, and the communication partner in real environments [104].

### 6.3. Mobile APP Integration and Mobile Health Applications

The development of AAC APPs utilizing the capabilities of smart phones and tablets could also be further explored to assist the communication of individuals with a speech disability, in turn reducing cost and enhancing portability. As demonstrated, the usage of VOCAs is of a potential benefit for users of different age ranges suffering a variety of medical conditions [112]. However, it is shown that the variability in terms of operational principles, user groups, and the complexity of a real and complicated user’s environment still need to be tackled. The implications for future research could also expand beyond the usage of the AAC devices for simple communication, as the integration of high-tech AAC with accessible smart devices paves the way for state-of-the-art developments, such as mobile health (m-Health) communicative applications, to exist. The development of smart mobile platforms would in turn aid the remote communication between users and their medical practitioners. This would expand the scope of AAC beyond physical communications, increasing the usability and the context of usage of future AAC solutions.

## Figures and Tables

**Table 1:** Sensing modalities of AAC signals.

Signal Sensing Category	Activation Method
Imaging methods	Eye gaze systems, head-pointing devices
Mechanical and Electromechanical methods	Mechanical keyboards, switch access
Touch-activated methods	Touchscreens, touch membrane keyboards
Breath-activated methods	Microphones, low-pressure sensors
Brain–Computer Interface methods	Invasive and non-invasive

**Table 2:** Signal sources, areas of strength and areas of limitation of current commercial AAC devices.

Signal Source	Mode	Typical Hardware Requirements	Areas of Strength	Limitations and Areas of Improvement
Imaging methods	Eye gazing	IR/NIR illumination source (commonly: 850 +/− 30 nm) Monitor Camera	Non-invasive Minimal voluntary control of muscles Can be used with patients requiring mechanical ventilation [26] IR is invisible to the user’s eyes [23] IR can stabilize gaze estimation [23]	High Temporal resolution = high volume of data as patterns are averaged over long time spans [101]; Consequence: sample sizes are often small [102] Eye tracking data processing [102] and parsing is complex [101] Need for calibration algorithms IR signals are not reliable for outdoor use [23] Generally, high cost [27]
	Head-pointing	Head-mounted visors in addition to a monitor and a camera [106] Light/optical pointers [4,19]	Less expensive compared to typical eye-gaze systems	Need fine user precision and controls [106] In direct contact with the user’s head
Mechanical/Electro- mechanical methods	Automatic, step, or inverse activation	Single switch, array of switches	Requires minimal motor control [4]	Generally slow
	Typing/icon selection	Mechanical keyboards	Instant feedback to user whenever a key is pressed [4,19]	Voluntary muscle control is a requirement for activation [4,19]
Touch-activated methods	Force production through: Hand/arm/ body part control Control extender [107]	Resistive/Capacitive touch screen circuitry Membrane keyboards: Non-conductive spacers separating conductive flat surfaces [4,19]	Minimal activation pressure	No direct feedback upon activation Requires appended feedback mechanisms (auditory/sensory) [4,19]
Breath-activated methods	Fine breath tuning [58]	Thoracic belt Sensor measuring the changes of waist circumference	Integration with a predictive language model	Physical control of movements: restricted for paralyzed users. Portability constraints Slow conversational rate
	Sniff control [59]	Control sensors for the acquisition of nasal pressure.	Confirmed usability with patients in LIS	Slowness: rate of three characters per minute.
	Discrete breath encoding [61,108]	Microphones/MEMS sensors Microcontroller boards	Wearable configuration	Digitized inputs Predefined words and sentences (not user-selected) Confinement to limited patterns.
	Analogue breath encoding [57]	Microphone PC	Continuous/analogue breath encoding	The processing of warped distances is computationally complex Portability constraints
BCI methods	Invasive	Implantable electrodes	Communication and control of environment without the need for body movements [64]	Prone to classification errors [22] Low transfer rates of ECG-based BCI due to the low signal to noise ratio [68] Most platforms are not yet suitable for everyday usage/ in-home usage BCI devices often require extensive assistance from caregivers [109]
	Non-invasive	External monitoring: EEG, MEG, fMRI, NIRS. [63]		

## Abbreviations

The following abbreviations are used in this manuscript:

AAC	augmentative and alternative communication
AI	artificial intelligence
ALS	Amyotrophic Lateral Sclerosis
APP	applications
AR	auto-regressive
ASD	Autistic Spectrum Disorders
AT	assistive technology
BCI	brain computer interface
DL	deep learning
EEG	electroencephalogram
fMRI	functional magnetic resonance imaging
HAAT	Human Activity Assistive Technology
IR	Infrared
IR-PCR	Infrared pupil corneal reflection
LIS	Locked-in-Syndrome
MEG	magnetoencephalography
MEMS	micro-electro-mechanical systems
ML	machine learning
MSE	mean square error
NIR	near-infrared
NIRS	near-infrared spectroscopy
NLP	natural language processing
PPG	photoplethysmogram
SGD	speech generating device
SLCN	speech, language or communication need
SMSW	synthesized machine spoken words
VOCA	voice output communication aid
WPM	words per minute
